# Medical education in a foreign language and history-taking in the native language in Lebanon – a nationwide survey

**DOI:** 10.1186/s12909-016-0826-7

**Published:** 2016-11-22

**Authors:** Vanda Abi Raad, Kareem Raad, Yazan Daaboul, Serge Korjian, Nadia Asmar, Mouin Jammal, Sola Aoun Bahous

**Affiliations:** 1 Gilbert and Rose-Marie Chagoury School of Medicine, Lebanese American University, Byblos-Jbeil, Lebanon; 2 Lebanese American University School of Medicine, Lebanese American University Medical Center - Rizk Hospital, May Zahhar Street, P.O. Box: 11-3288, Ashrafieh, Beirut Lebanon; 3 School of Public Health, University of California, Berkeley, CA USA; 4 Department of Medicine, Tufts Medical Center, Boston, MA USA; 5 Department of Medicine, Beth Israel Deaconess Medical Center, Harvard Medical School, Boston, MA USA; 6 Faculty of Medicine, Saint-Joseph University, Beirut, Lebanon

**Keywords:** Medical education, History-taking, Native language, Self-reported confidence level, Survey

## Abstract

**Background:**

With the adoption of the English language in medical education, a gap in clinical communication may develop in countries where the native language is different from the language of medical education. This study investigates the association between medical education in a foreign language and students’ confidence in their history-taking skills in their native language.

**Methods:**

This cross-sectional study consisted of a 17-question survey among medical students in clinical clerkships of Lebanese medical schools. The relationship between the language of medical education and confidence in conducting a medical history in Arabic (the native language) was evaluated (*n* = 457).

**Results:**

The majority (88.5%) of students whose native language was Arabic were confident they could conduct a medical history in Arabic. Among participants enrolled in the first clinical year, high confidence in Arabic history-taking was independently associated with Arabic being the native language and with conducting medical history in Arabic either in the pre-clinical years or during extracurricular activities. Among students in their second clinical year, however, these factors were not associated with confidence levels.

**Conclusions:**

Despite having their medical education in a foreign language, the majority of students in Lebanese medical schools are confident in conducting a medical history in their native language.

## Background

Effective doctor-patient communication is essential for delivering quality healthcare [1–5]. Nevertheless, language barriers remain some of the most reported hindrances that physicians need to overcome in a healthcare setting [6–8]. While approximately one third of international medical schools outside the United States and Canada offer education in English, only one-fifth of the countries, within which these institutions are located, list English as an official language [9]. The broad adoption of the English language in medical education largely follows the international medical community that has adopted English as the official language for the majority of medical journals and international conferences and allows greater access to specialty training in English-speaking countries [10, 11].

Lebanon is a good example of the divergence of spoken and educational languages. Out of the seven registered medical schools in the country, four use English as their language of medical instruction, while the remaining use French; two languages that are not primary speaking languages in the country. The official and most widely spoken language in Lebanon is Arabic, and the majority of patients in academic and community healthcare centers speak it. Lebanon is also unique in having a high proportion of bilingual and trilingual medical students [12]. Both these factors create a unique population of medical students for evaluating the influence of medical education in a foreign language on patient communication in the native language. The present study investigates the association between medical education in a foreign language and medical students’ self-perceived confidence in their history-taking skills in their native language.

## Methods

### Participants

All registered medical schools in Lebanon were invited to participate (*n* = 7). Students rotating in clinical clerkships were included (defined as either Med III or Med IV in schools adopting the American model or Med VI or Med VII in schools adopting the French model). In brief, 3 medical schools incorporate the American model (4-year program), 2 incorporate the French model (7-year program), and 1 incorporates the British model (6-year program). English is the language of instruction in schools that follow the American and British models (*n* = 4), whereas French is the language of instruction in schools that follow the French model (*n* = 3). Medical schools of the American model include 2 years of pre-clinical education followed by 2 years devoted for clinical rotations. In contrast, medical schools adopting the French or the British model typically devote 1 or 2 years of clinical observerships followed by 2 final years of clinical training.

### Ethics, consent, and permissions

All participants were informed that their participation was voluntary. The study objective was explained to all participants, and verbal consent was obtained. All in-person and online surveys were anonymized and untraceable. No individual subject identifiers were collected. The study protocol was approved for exemption by the local institutional review board a the Lebanese American University and was in accordance with the Helsinki Declaration and its later amendments.

### Survey

Students were invited to anonymously fill a standardized 17-question survey. Surveys were administered in a mixed-format approach either in-person or using an online tool based on the requirements for surveys per medical school over a 4-month data collection period. The survey was developed based on a prior study and focused on the linguistic aspects of the students’ education and daily life, as well as their self-perceived confidence in their history-taking skills (Table 1) [13]. Questions in the survey were either open-ended, categorical, or ordinal. Open-ended questions addressed the spoken language at home and languages of high school, undergraduate, and medical education. Categorical questions addressed academic level, gender, Arabic as native language, bilinguality, and pre-clinical curricular or extracurricular exposure to conducting a medical history in Arabic. Ordinal questions captured students’ rating on a 4-point Likert scale, which addressed confidence levels in conducting medical histories and the perceived impact of communication courses on communication skills. The Likert scale points were equidistant and included 1-not at all, 2-a little, 3-moderate, and 4-very. An initial version of the survey was piloted among a subgroup of Lebanese medical students, and questions were either added or modified based on subject feedback.

**Table 1:** Survey tool

Question	Answer Choice Type
Academic level (clinical year 1 vs. clinical year 2)	Binary Categorical
Gender	Binary Categorical
Is Arabic your primary language?	Binary Categorical
Were you raised bilingual?	Binary Categorical
If yes, which languages did you speak at home as a child?	Open-Ended Short Answer
High school language	Open-Ended Short Answer
Undergraduate language	Open-Ended Short Answer
Medical instruction language	Open-Ended Short Answer
Which language did you speak yesterday at dinner?	Open-Ended Short Answer
Have you had any practice in history taking/communication skills in Arabic during your clinical skills courses in the pre-clinical years, before you began working in a hospital?	Binary Categorical
Have you practiced history taking or medical communication skills in Arabic outside the hospital (e.g. Red Cross)?	Binary Categorical
Do you feel confident in taking a history in French or English?	Likert Scale (4 Categories)
Do you feel confident taking a history in Arabic?	Likert Scale (4 Categories)
Will most of the patients you will see in the future speak Arabic/English/French/Other?	Categorical
For those who presented Objective Structured Clinical Examinations (OSCEs) during the pre-clinical years in English/French instead of Arabic, do you think this negatively affected your communication skills in Arabic at the hospital?	Likert Scale (4 Categories)
Do you think it would be beneficial to introduce a communication course in Arabic before you begin working in a hospital setting?	Likert Scale (4 Categories)
Do you have any suggestions to improve the communication skills of Lebanese medical students in Arabic?	Open-Ended Short Answer

### Statistical analysis

All statistical analyses were performed using Stata® 14 (StataCorp LP, TX, USA). Data was analyzed for all subjects who had an available answer for the outcome (i.e. confidence level in conducting a medical history in Arabic). Characteristics of the study population were evaluated using descriptive statistics. Ordinal variables of interest, including levels of confidence in conducting a medical history in Arabic, were first recorded as 4 categories according to the Likert scale, and were then dichotomized for subsequent analyses. The association between categorical variables was performed using Chi-square test. Cochran-Mantel-Haenszel test was performed to evaluate the association between categorical variables when stratification by clinical year was conducted. To evaluate the odds and independent association with the outcome in each clinical year, mixed-effects logistic regression was performed. Logistic regression models were fit via maximum likelihood and were clustered at the school level (i.e. random school effect) to account for the correlated outcomes for students within the same medical school. All variables that were associated with the outcome on univariate analysis with a *p*-value ≤ 0.10, as well as variables of historical interest, were included in the logistic regression models. All *p*-values were two-sided. A *p*-value < 0.05 was considered statistically significant.

## Results

A total of 1,021 medical students from all medical schools in Lebanon were invited to participate in the study, of which 458 responded (response rate = 44.9%) and 457 were eligible for the final analysis (analysis population = 99.8% of responders). Participation was similar across both genders (males 52.7% vs. females 47.3%), and the majority of participants were enrolled in their first clinical year (65.0% vs. 35.0%). Arabic was the native language for 91.6% of participants. Compared with the second clinical year, there were significantly more participants in their first clinical year who had English as their language of medical education (65.0% vs. 51.9%, *p* = 0.006). Table 2 summarizes the characteristics of the study participants.

**Table 2:** Characteristics of the study population

Characteristic	Clinical Year		*p*-value
	CY 1 (*N* = 297)	CY 2 (*N* = 160)	
Male gender, % (n)	52.9% (157)	52.5% (84)	0.94
Arabic native language, % (n)	91.2% (271)	92.4% (145)	0.68
Medical school model English model, % (n) French model, % (n) Both, % (n)	65.0% (193) 22.9% (68) 12.1% (36)	51.9% (83) 18.1% (29) 30.0% (48)	<0.001
Language of medical education English, % (n) French, % (n)	65.0% (193) 35.0% (104)	51.9% (83) 48.1% (77)	0.006
Exposure to Arabic history-taking courses in pre-clinical years, % (n)	44.7% (131)	49.0% (77)	0.38
Arabic history-taking in extracurricular activities, % (n)	25.9% (77)	24.4% (39)	0.72
In-person method of distribution, % (n)	49.5 (147)	55.0% (88)	0.26

*Note*: Percentages were calculated for subjects with available data

*Abbreviation*: *CY* Clinical year

Although all participants had their medical education in a foreign language, 88.5% of participants whose native language was Arabic were either extremely or moderately confident that they could conduct a medical history in Arabic during their clinical years (first clinical year: 87.5% vs. second clinical year: 90.3%, *p* = 0.38). In contrast, this proportion was significantly lower among participants whose native language was not Arabic (64.9%, *p* < 0.001). When further subgrouped by clinical year, this difference remained statistically significant among students in their first clinical year (87.5% vs. 57.7%, *p* < 0.001), but was not significant when participants in the second clinical year were evaluated alone (90.3% vs. 81.8%, *p* = 0.37). Despite varying results between both clinical years, clinical year did not significantly modulate the effect of Arabic being the native language on Arabic history-taking confidence levels (p-interaction = 0.23).

To evaluate the factors that were independently associated with high confidence levels in conducting a medical history in Arabic in each clinical year, a separate multiple logistic regression model was constructed for each clinical year. Among participants enrolled in the first clinical year, high confidence in Arabic history-taking was independently associated with Arabic being the student’s native language and exposure to Arabic history-taking in either pre-clinical years or during extracurricular activities. In contrast, the impact of all these factors on confidence levels was lost when participants enrolled in the second clinical year were evaluated alone (Table 3).

**Table 3:** Univariate and multivariate associations with confidence in conducting a medical history in Arabic

Variable	Confidence in conducting a medical history in Arabic (outcome)			
	Univariate OR (95% CI) *p*-value		Multivariate OR (95% CI) *p*-value	
	CY1	CY2	CY1	CY2
Male gender	1.1 (0.6, 2.2) *p* = 0.75	1.7 (0.6, 4.8) *p* = 0.31	-	-
Arabic native language	9.3 (3.3, 26.1) *p* < 0.001	2.1 (0.4, 11.3) *p* = 0.37	15.3 (4.8, 49.0) *p* < 0.001	3.7 (0.6, 23.0) *p* = 0.16
English medical education	3.0 (0.6, 14.3) *p* = 0.16	2.8 (1.0, 8.5) *p* = 0.06	3.6 (0.6, 21.0) *p* = 0.15	3.5 (0.99, 12.0) *p* = 0.051
Exposure to Arabic history-taking courses in pre-clinical years	6.7 (2.5, 17.5) *p* < 0.001	2.5 (0.8, 7.5) *p* = 0.10	8.7 (2.8, 26.3) *p* < 0.001	2.1 (0.7, 6.7) *p* = 0.19
Arabic history-taking during extracurricular activities	3.9 (1.4, 11.1) *p* = 0.01	N/A^a^	5.6 (1.6, 20.0) *p* = 0.007	-
High confidence in English/French history taking	1.3 (0.4, 4.1) *p* = 0.65	0.4 (0.1, 3.0) *p* = 0.36	1.7 (0.4, 6.2) *p* = 0.45	0.6 (0.1, 5.3) *p* = 0.71

*Abbreviations*: *CI* Confidence interval, *CY* Clinical year, *OR* Odds ratio

^a^Odds ratio cannot be calculated since all participants (100%) who reported a positive response also reported a positive outcome

Interestingly, confidence in conducting a medical history in Arabic was irrespective of the participants’ confidence in history-taking in the foreign language of medical education. Approximately 87% of participants who were not confident in conducting English/French history-taking were still confident in conducting a medical history in Arabic. This difference was consistent across both clinical years (first clinical year: 80% vs. second clinical year: 95.2%).

**Fig. 1:**
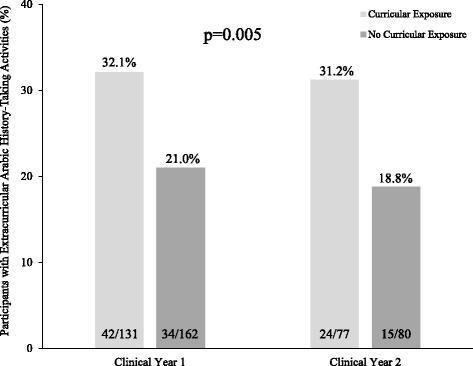
Association between curricular exposure to Arabic history-taking during pre-clinical years and involvement in extracurricular activities that require Arabic history-taking

Subsequent analysis demonstrated that when stratified by clinical year, pre-clinical curricular exposure to Arabic history-taking was associated with a higher likelihood of a student being engaged in extracurricular activities involving Arabic history-taking (e.g. volunteer services) (first clinical year: 32.1% vs. 21.0%; second clinical year: 31.2% vs. 18.8%, *p* = 0.005) (Fig. 1). Although the vast majority of participants (82.9%) believe their future patients will speak Arabic, only 64.5% of those perceived that adding courses for communication in Arabic will be beneficial (first clinical year: 66.4% vs. second clinical year 61.1%, *p* = 0.31), and only 29.1% of them perceived that having an Objective Structural Clinical Examination (OSCE) in a foreign language may have negatively affected their communication in Arabic during the clinical years (first clinical year: 28.7% vs. second clinical year 29.7%, *p* = 0.86).

## Discussion

This study demonstrates that during the clinical years, the vast majority (88.5%) of medical students in Lebanon are confident in conducting a medical history in their native language (Arabic) despite having their medical education a foreign language (in either English or French). This proportion was substantially higher in this study as compared with other populations, where confidence levels have previously been reported to be as low as 30% [13]. Although the exact etiology of this contradistinction is unclear, it may be attributed, at least in part, to the unique bilingual nature of the Lebanese population in general, which is not universally common among other studied populations [13–16].

This study further highlights that effective doctor-patient communication among medical students is largely dependent on their exposure to real-life patients in the hospital setting. Although higher confidence level in Arabic history-taking during the first clinical year was associated with Arabic being the native language, as well as prior curricular and extracurricular exposure to Arabic history-taking, this association was evidently not present among students in their second clinical year. Although the clinical year did not significantly modulate the effect of Arabic being the native language on Arabic history-taking confidence levels, the results from Table 3 suggest that as medical students transition from their first clinical year to their second clinical year, the impact of external factors becomes less relevant in predicting confidence in history-taking skills in their native language. These findings are consistent with prior studies, where medical students perceived that a foreign language of medical instruction may be an obstacle only during the first year of medical school, but was no longer important during the subsequent medical years [17]. Accordingly, this study demonstrates that in Lebanon, medical education in a foreign language does not represent a gap in effective communication between graduating medical students and their patients.

This is the largest representative study among medical students to correlate medical education in a foreign language to the confidence in conducting a medical history in the native language. Nonetheless, this study is limited by its cross-sectional nature and differences in the sample sizes and characteristics between first and second clinical year students. Although the overall response rate was lower than the majority of in-person surveys, it was higher than most online nationwide surveys conducted among medical students. Finally, the unique characteristics of the Lebanese population, i.e. the majority of whom are raised bilingually, limits the generalizability of the results to other populations.

## Conclusions

Despite receiving their medical education in a foreign language, the majority of students in Lebanese medical schools report being confident in conducting a medical history in their native language. In the first clinical year, high confidence levels in conducting a medical history in Arabic was associated with having Arabic as the student’s native language, as well as engagement in Arabic history-taking in the pre-clinical years and during extracurricular activities. These factors were not associated with the students’ confidence in the second clinical year, suggesting that the experience accrued in the first clinical year overcomes the external elements that may limit their confidence in conducting a medical history in their native language.

## Abbreviations

OSCE: Objective structural clinical examination
